# Syphilis in Infancy and Early Life

**Published:** 1911-11

**Authors:** George K. Varden

**Affiliations:** Atlanta, Ga.


### SYPHILIS IN INFANCY AND EARLY LIFE.

By George K. Varden, M. D., Atlanta, Ga.

For the most part we shall deal with the disease as seen in the early months of life, touching only lightly upon manifestations encountered later on.

The diagnosis and treatment concern us most and to these our energies will be directed.

The Spirochaeta Pallida is, in all probability the true cause of the disease, since it occurs in the lesions and inoculation experiments on animal produce syphilis.

It is upon the eruption more than any symptom that diagnosis rests and this eruption I have never seen at birth.

The disease most frequently starts with a coryza commonly called "snuffles," and some huskiness of the crying voice. The nasal discharge may be clear or mixed with blood and this ushers in the disease, usually in the first three months of life. Crusts form in the nose and mouth breathing is induced which makes nursing difficult.

The eruption most frequently seen has been maculo-papular, occurring on the extensor surfaces of the arms and legs, the mid-portion of face, back of neck, buttocks, perineal region and inner parts of thighs. The flexor surface of wrists and ankles are frequently the seat of macules or papules. The palms of hands and soles of feet are similarly involved but not so frequently, the commoner condition being a slight scaliness. The macules, papules or combined eruption is brownish red in color, situated on a reddened base, and soon after appearing begin to lose the red blush and fine scales are seen capping the lesions: the duration of the eruption is rarely more than three weeks though other symptoms persist. In size these macules or papules approach that of the infant's nails, but this is variable, yet they do not approach the size of the larger papular syphilide as seen in the adult. Occasionally a pemphigoid eruption has been associated with the one described, but rarely alone.

Some writers have called attention to an unusually heavy crop of hair in syphilitic infants, which is most frequently dark in color and to this has been given the name "syphilitic wig." Later on this is followed by a general thinning of the hair which is quite unlike an Alepecia Areata.

Two points in particular have attracted my attention in these children. The first is the rather queer appearance of the upper part of the face: it is probably due to the thinness of the eyebrows and lashes. They look bare, and as though they had been greased on either side of the nose, about the eyes, and the lower part of the forehead. This greasy appearance may be accounted for by an over activity of the sweat or sebaceous glands.

The second point of interest is a peculiar waxy appearance of the palms and soles. The color here is normal, but the skin looks as if it had been covered by a very thin layer of paraffin paper—the wrinkles in the skin corresponding with the cracks in the wax produced by folding of such paper.

One or more fingers or toes may be reddened at the distal portion and nail may be freed in part or cast off and this constitutes the specific enychia. This must not be confused with the simple redness produced by finger sucking.

Fissures may be found at corners of mouth and on opening mouth a white line may be seen extending backward on mucous membrane for a short distance.

Mucous patches of mouth and throat are not seen so frequently as in adults and less often in the negro than in the white.

In those of ten to sixteen months of age scattered sqaumous papules which later have a smooth, flat top of a pinkish or violaceous hue are to be seen about the wrists and ankles, both surfaces of the arms, on the hips and occasionally on the anterior surface of the tibia. These are usually few in number but are very characteristic.

The absence of enlarged glands in early syphilis is explained by Edmunds, who states that lymphatics in man are not developed fully until the fifth month of life.

General nutrition in this disease is better than most accounts would lead us to believe, though if untreated or artificially fed wasting is marked and prognosis becomes grave.

Enlarged liver and spleen are often associated with other conditions at this time.

Cranio-tabes in all probability has no connection with lues.

Syphilitic epiphysitis and esteo-periostitis I have seen but never earlier than the fifth month, though both occur earlier. Hutchinsonian teeth, iritis, interstitial keratitis, and sunken nose belong to late syphilis.

Acquired syphilis I have not seen in infancy or early childhood, though cases with tardy eruption may be very confusing, as the skin is not involved until long after the rash of congenital disease, which occurs as has already been stated, in the first three months of life.

Three conditions of the skin must be excluded before making the diagnosis of syphilis and I shall name them in order of frequency of occurrence: I. Erythema of napkin region, Impetigo Bullosa and Scabies which in young children is different from the disease in the adult.

Erythema may be as the name implies only an erythematous blush or it may have with it macules, papules or vesicles, these occurring in combination or separately. This skin lesion is limited to the convex surfaces, as the buttocks, heels, and calves of legs, as these parts are in contact with the wet napkins.

The fact that the color changes from day to day is rather characteristic of the erythema and is probably due to wet napkins in part; uncleanliness is a big fatcor in the causation of the disease. The perineum is sometimes involved as a result of the drawing up of the napkin.

Bullous Impetigo resembles a bleb as produced by a burn: rupture of one or more blebs takes place and this dries to form a crust, the color of such crust depending upon whether the content is clear, pustular or haemorrhagic. Location is oftenest on the face or hands and the leg from knee to ankle is not infrequently the site of the lesion described.

Scabies when infected by scratching with dirty hands and nails produces about the wrists suspicious sores at times and these are confusing. The burrow of the mite must be sought between the fingers, the eruption occurs on the face in the very young while it is free in the adult, itching is or has been intense and there is history of other members of the family having had a skin eruption which was made unbearable by a constant desire to scratch and this desire gratified in anyone's society.

This covers the diagnosis of the skin diseases most frequently causing doubt.

Adenoids do occur in infants during the first few months of life and give rise to a more or less chronic nasal discharge: this discharge I have seen attributed to syphilis when no other sign than feeble development was present with it.

Examination of naso-pharynx would have prevented the error.

Enlarged glands are discoverable in many malnourished children who show nothing else suggestive of the disease under consideration: a general adenopathy is too frequently interpreted as a specific anifestation and drug treatment unnecessarily instituted.

TREATMENT.

Extreme care must be exercised in handling a syphilitic infant, all utensils used either by or for them should be kept away from other persons, old or young.

Authorites differ as to the danger of contracting the disease from a case of congenital lues. Some urge that it is very contagious and go even further, stating that there is an indefinitely greater power of contagion than in the acquired form. There are others who are equally capable who state they have never seen it so spread.

However this may be, we should never handle a case with fissures and other secondary lesions except with extreme care, taking precaution to scrub up well afterwards.

The management of the feeding is very important and what has been said in favor of breast feeding as opposed to hand feeding in infancy applies with greater force in syphilis if a successful outcome is to be expected. This, of course, is to be done by the mother and never by a wet nurse.

Mercury is the drug that offers the best result and the mode of administration is the point to be determined. Grey powder is the form which I prefer early and this can be given in doses ranging from 1-2 to 1 gr. three times a day. This can be continued for a year at any rate.

Should the nature of the case demand that patient be gotten under the influence in a short time, this can be supplemented by daily injections of mercurial ointment.

Too frequent stools are unusual when Grey Powder is administered and it has the further advantage of being more cleanly than injections.

I have used Ammoniated Mercury ointment as an inunction and like it.

Salvarsan I have had no opportunity to employ as yet, but would certainly start with a smaller dose than has been employed by some that I might avoid accidents such as have been reported from the use of the drug in the young.

Most interesting are the reports of cases where the drug has been given to the mother who continued to nurse her child with appreciable improvement of all symptoms.

In the latter months of the disease I have used mixed treatment, that is, bichloride of mercury and potassium iodide in watery solution with enough syr. saparilla comp. to disguise in a measure the unpleasant taste.

Results obtained have been quite good and gastro-intestinal symptoms were wanting.

I cannot refrain from condemning the use of the iodides alone as a routine practice early and late: while it is true that symptoms and signs disappear with its use, it is equally true that they do so only to reappear and this is much more infrequent with the use of mercury or the combined use of the drugs.

For the late lesions their use is justified but it is my belief that in most instances these could have been prevented by more active early treatment.

